# Neural circuit analysis using a novel intersectional split intein-mediated split-Cre recombinase system

**DOI:** 10.1186/s13041-020-00640-2

**Published:** 2020-07-02

**Authors:** Audrey Tze Ting Khoo, Paul Jong Kim, Ho Min Kim, H. Shawn Je

**Affiliations:** 1 Neuroscience and Behavioural Disorders Programme, Duke-National University of Singapore (NUS) Medical School, 8 College Road, Singapore, 169857 Singapore; 2 Graduate School of Medical Science & Engineering, Korea Advanced Institute of Science and Technology (KAIST), Daejeon, 34141 Republic of Korea; 3 Center for Biomolecular & Cellular Structure, Institute for Basic Science (IBS), Daejeon, 34126 Republic of Korea

**Keywords:** Split intein, Split-cre recombinase, Circuit mapping, Projection neurons, Retrograde virus, DLX6, GABA, Transgenic mouse

## Abstract

The defining features of a neuron are its functional and anatomical connections with thousands of other neurons in the brain. Together, these neurons form functional networks that direct animal behavior. Current approaches that allow the interrogation of specific populations of neurons and neural circuits rely heavily on targeting their gene expression profiles or connectivity. However, these approaches are often unable to delineate specific neuronal populations. Here, we developed a novel intersectional split intein-mediated split-Cre recombinase system that can selectively label specific types of neurons based on their gene expression profiles and structural connectivity. We developed this system by splitting Cre recombinase into two fragments with evolved split inteins and subsequently expressed one fragment under the influence of a cell type-specific promoter in a transgenic animal, and delivered the other fragment via retrograde viral gene transfer. This approach results in the reconstitution of Cre recombinase in only specific population of neurons projecting from a specific brain region or in those of a specific neuronal type. Taken together, our split intein-based split-Cre system will be useful for sophisticated characterization of mammalian brain circuits.

## Introduction

The human brain is arguably the most complex and sophisticated organ made up of intricate networks of neurons connected to each other across different brain regions. These neuronal networks process external stimuli, produce sensory and emotional experiences to generate calculated responses to the environmental stimuli based on intrinsic mechanisms or learned experiences [21, 25, 29]. A fundamental goal in neuroscience therefore is to understand the functions of individual neurons and to delineate how different groups of neurons function together to orchestrate animal behavior [35]. To achieve this goal, researchers have utilized molecular and genetic tools in model organisms using binary expression systems, such as the Cre-LoxP system in mice and the GAL4-UAS system in *Drosophila* [2, 31]. Coupled with recent technological advances, including optogenetics to manipulate neuronal activity, voltage- or calcium-sensing proteins to visualize activity, and genetically engineered animals, these binary expression systems have tremendously expanded our knowledge of certain neuronal populations [31, 34].

Although the Cre-LoxP system has been vital in helping us understand neuronal function, single gene expression profile is often insufficient to target specific neuronal populations of interest [6, 10]. For example, recent results from single-cell RNA sequencing analyses indicate high levels of transcriptomic heterogeneity among neurons of the same subtype, even within the same brain subregions [13]. As such, intersectional bipartite systems were developed to target more genetically defined and homogenous populations of neurons. Hirrlinger et al. [6] utilized a Cre-based complementation system by splitting Cre recombinase into two complementation-competent Cre protein fragments, allowing expression of the two split-Cre fragments to be driven by two different promoters. This strategy limited the expression of Cre recombinase to only cells that expressed both fragments of Cre [6]. Similarly, Jullien et al. [9] split Cre recombinase into two complementary-competent fragments that could be reconstituted via a ligand to achieve better temporal control over the expression of Cre recombinase. Despite their utilities, these split-Cre systems are difficult to implement under conditions that require continuous cellular expression of Cre recombinase as they require exogenous ligands for recombination activity [37]. Here, we present a novel method to reconstitute split-Cre recombinase using split inteins, which are self-catalytic protein elements that facilitate protein trans-splicing reactions [18, 24, 27], to overcome the need for exogenous ligands to reconstitute Cre recombinase. By using this split intein-mediated split-Cre recombinase system, we aimed to label long-range GABAergic projection neurons that could not be genetically targeted with the current research tools [8, 15, 20, 22]. By simply expressing one split-Cre fragment in the neurons of the GABAergic lineage and delivering the other fragment via retrograde viral gene transfer, we were able to constitute Cre activity in only long-range GABAergic neurons that projected their axons from the central amygdala (CeA) to the dorsal striatum (DS).

## Results

**Fig. 1:**
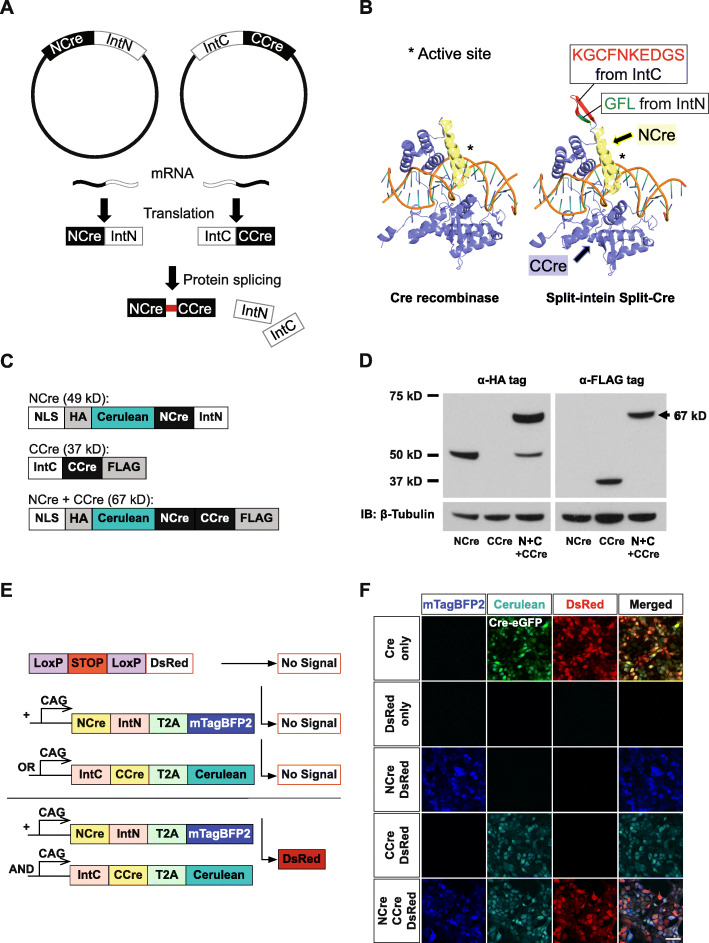
Design of the split intein-mediated split-Cre recombinase system. **a** Schematic depicting the autocatalytic split intein-mediated trans-splicing reaction to reconstitute Cre recombinase. Split inteins associate to fuse NCre and CCre with a peptide bond. **b** Structural comparison of original Cre recombinase-LoxP DNA complex (left, PDB ID: 1NZB [5] with a model for split intein-mediated split-Cre recombinase-LoxP DNA complex (right). The structure of split inteins from IntN (GFL) and IntC (KGCFNKEDGS), NCre and CCre are colored as green, red, yellow and blue respectively. Conserved active site are marked with an asterisk. **c** Schematic demonstrating the predicted size of the protein when NCre-IntN tagged with HA (49 kD) or IntC-CCre tagged with FLAG (37 kD) are transfected alone. A 67 kD reconstituted product is expected when both NCre-IntN and IntC-CCre are cotransfected. **d** Western blot analysis showing that when NCre-IntN is transfected alone, a 37 kD protein band was observed when western blotting was performed using a specific antibody against HA. When IntC-CCre was transfected alone, a 49 kD protein band was observed when western blotting was performed using a specific antibody against FLAG. When NCre-IntN and IntC-CCre were cotransfected, a 67 kD protein band with a higher molecular weight was observed when western blotting was performed using a specific antibody against HA or FLAG. These results indicated that the reconstituted 67 kD product comprised of components from both NCre-IntN and IntC-CCre. **e** Design of the in vitro reporter assay. HEK293T cells were transfected with NCre-IntN or IntC-CCre alone or together with a LoxP-Stop-LoxP-DsRed reporter. **f** When NCre-IntN or IntC-CCre were transfected alone, no DsRed expression was observed. When NCre-IntN and IntC-CCre were cotransfected along with the reporter in HEK293T cells, we observed strong DsRed expression. Scale bar, 50 μm

We split Cre recombinase into two fragments, NCre (aa1–59) and CCre (aa60–343) [10], and attached an N- or C-intein of the evolved split intein Npu37, which can express and trans-splice efficiently in mammalian cells [18], to the C-terminal sequence of NCre and the N-terminal sequence of CCre respectively. This resulted in two fusion genes: NCre-IntN and IntC-CCre (Fig. 1a). The transcription and translation of the NCre-IntN and IntC-CCre fusion genes lead to the binding and autocatalytic trans-splicing of the split inteins. This reaction then ligates NCre and CCre together via peptide bonds to form functional Cre recombinase with additional peptide sequences (KGCFNKEDGS from IntC and GFL from IntN) [26]. Based on our 3D modeling, the additionally incorporated peptide sequences do not occlude the active DNA-binding site of the reconstituted Cre recombinase (Fig. 1b). To test whether split intein-mediated trans-splicing could occur in mammalian cells under physiological conditions, we expressed epitope-tagged NCre-IntN and IntC-CCre constructs in HEK293T cells, and subjected the resulting lysates to western blot analysis (Fig. 1c and d). When either NCre-IntN or IntC-CCre was transfected into HEK293T cells alone, only a single 49 kD band or a 37 kD band was observed by western blot using specific antibodies against either HA or FLAG respectively (Fig. 1d). However, when both NCre-IntN and IntC-CCre were transfected, we observed a band of higher molecular weight (67kD) detectable by both HA and FLAG specific antibodies, indicating successful split intein-mediated trans-splicing reaction under physiological conditions (Fig. 1d).

**Fig. 2:**
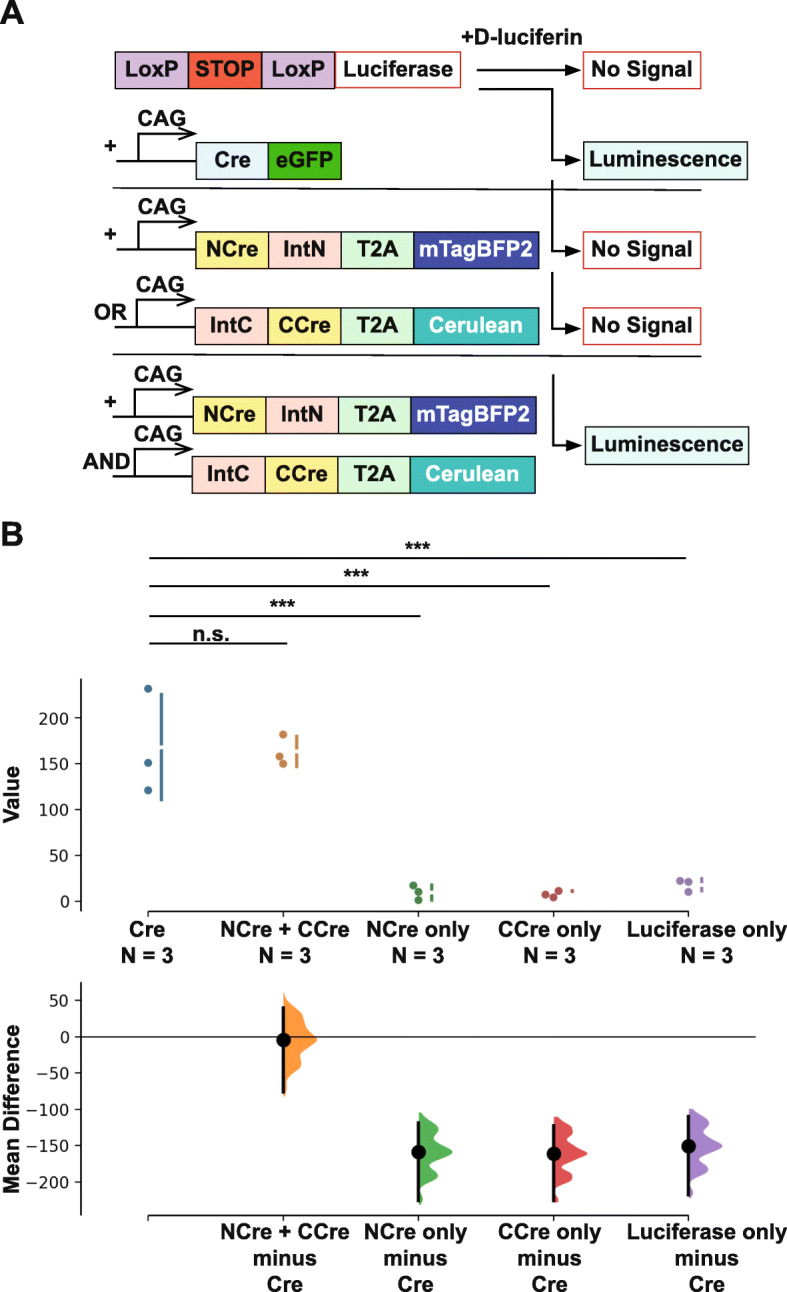
Design of luciferase assay. HEK293T cells were transfected with Cre recombinase, NCre-IntN or IntC-CCre alone, or together, with a LoxP-stop-LoxP-Luciferase reporter. **a** Schematic showing the experimental design for transfecting cDNA constructs in HEK293T cells. **b** The Cre activity is defined by the amount of luminescence detected in the luciferase assay. The mean difference for 4 comparisons against the shared control Cre recombinase are shown in the above Cumming estimation plot. The raw data is plotted on the upper axes. On the lower axes, mean differences are plotted as bootstrap sampling distributions. Each mean difference is depicted as a dot. Each 95% confidence interval is indicated by the ends of the vertical error bars. Importantly, we observed similar levels of Cre activity for both the native Cre recombinase and the reconstituted split-Cre recombinase. * *p* < 0.05, ** *p* < 0.01, *** *p* < 0.001

Next, to validate whether the reconstituted Cre recombinase via trans-splicing of split inteins was functional, we attached fluorescent tags, mTagBFP2 and Cerulean to NCre-IntN and IntC-CCre respectively, to visualize the cellular expression of these constructs in HEK293T cells (Fig. 1e). We reasoned that the expression of these split-Cre constructs together with a LoxP-stop-LoxP DsRed reporter cDNA would easily reflect the activity and efficiency of split-Cre recombination through the presence of DsRed fluorescence in cells (Fig. 1e). As expected, we did not observe any DsRed fluorescence when either NCre-IntN or IntC-CCre was transfected alone. However, when both NCre-IntN and IntC-CCre were cotransfected, we observed strong DsRed signals, which was similar to cells transfected with the full-length Cre recombinase cDNA and reporter (Fig. 1f). We also observed that approximately 99% of the cells expressing both NCre-IntN and IntC-CCre exhibited DsRed fluorescence (Fig. 1f). Alternatively, we performed a luciferase assay as a readout for Cre-mediated recombination activity [11] by transfecting either NCre-IntN or IntC-CCre alone, or together into HEK293T cells expressing a LoxP-stop-LoxP luciferase cDNA construct (Fig. 2a). Twenty-four hours after transfection, we detected luminescence in only the cells cotransfected with both NCre-IntN and IntC-CCre (*p* < 0.001; Fig. 2b). Importantly, we observed that the Cre activities of reconstituted split intein split-Cre recombinase were comparable to that of native Cre recombinase (*p* = 0.798, no statistical significance; Fig. 2b [7]).

**Fig. 3:**
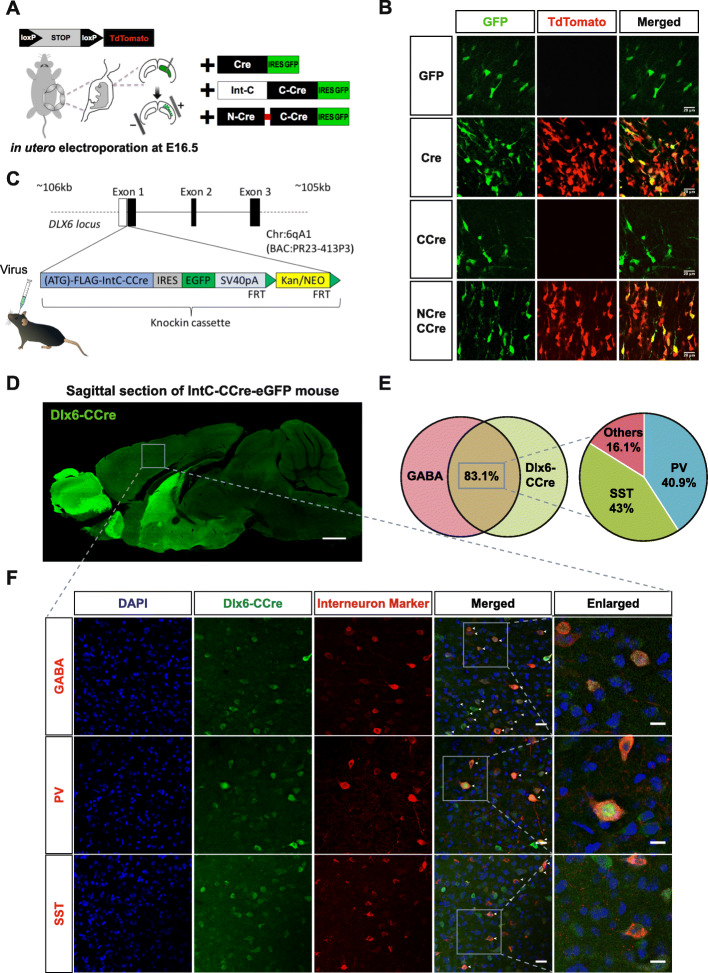
In vivo validation of the split intein-mediated split-Cre recombinase system. **a** Schematic showing the experimental design for electroporating constructs into LoxP-Stop-LoxP-TdTomato E16.5 embryos. **b** Strong TdTomato expression was observed with native Cre recombinase or when both NCre-IntN and IntC-CCre constructs were electroporated. No TdTomato was observed when only IntC-CCre was electroporated alone. This demonstrated that Cre-mediated recombination occurs only when both NCre and CCre are present. **c** Schematic diagram of BAC transgenic mice expressing IntC-CCre in forebrain GABAergic neurons. The resultant BAC transgenic mouse was then further crossed with the LoxP-Stop-LoxP-TdTomato reporter line mouse. Scale bar, 20 μm. **d** Sagittal section from a DLX-CCre-IRES-eGFP mouse showing that IntC-CCre-positive cells were predominantly found in the forebrain. Scale bar, 1 mm. **e** Venn diagram showing that 83.1% of DLX6-CCre neurons were GABA-positive. Of the CCre+/GABA+ neurons, 36.5% were PV and 41% were SST. **f** Immunohistochemical analyses showing DLX6-CCre-IRES-eGFP brain sections stained using specific antibodies against GABA, PV and SST. Scale bars, 20 μm (merged) and 10 μm (enlarged).

To test if the split-Cre system is functional in the in vivo mammalian brain, we delivered NCre-IntN and IntC-CCre, Cre recombinase, or GFP only constructs into the lateral ventricles of LoxP-stop-LoxP-TdTomato reporter mice on embryonic day 16.5 (E16.5) via in utero electroporation [36] (Fig. 3a). We did not observe any TdTomato expression when we electroporated GFP only or IntC-CCre fragment only (Fig. 3b). However, we observed strong TdTomato fluorescence in neurons when we electroporated both NCre-IntN and IntC-CCre constructs, and the fluorescence signal as well as a number of expressed neurons were similar to electroporation of native Cre recombinase (Fig. 3b). Taken together, these data showed that the reconstituted split intein-mediated split-Cre recombinase is functional in both in vitro and in vivo in mammalian brains.

To target long-range GABAergic projection neurons [30], we generated a transgenic mouse that expressed IntC-CCre-IRES-eGFP under the control of an endogenous *Dlx6* promoter known to be expressed exclusively in forebrain GABAergic neurons [1, 4, 14, 33] (Fig. 3c and d). Next, the resulting *Dlx6*-CCre-IRES-eGFP mice were further crossed with Rosa26-LoxP-stop-LoxP-TdTomato reporter mice [19] (Fig. 3d). Using immunohistochemistry, we confirmed that IntC-CCre expression was restricted to GABAergic neuronal populations (Fig. 3e and f). A total of 83.1% of the IntC-CCre neurons, indicated by green fluorescence, expressed GABA, and 40.9 and 43% of these neurons expressed PV and SST respectively (Fig. 3e and f).

**Fig. 4:**
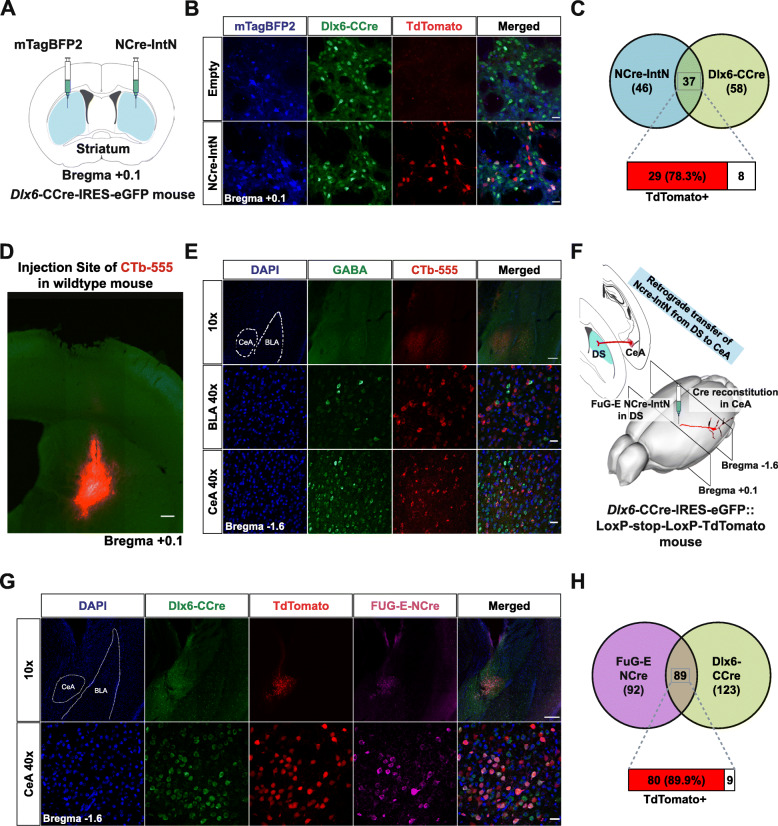
Targeting long-range GABAergic neurons using a DLX6-CCre-IRES-eGFP mouse and virus. **a** Schematic of stereotaxic injection of lentiviral particles to observe recombination of Cre recombinase in DLX6-CCre-IRES-eGFP mice. The mTagBFP2-only lentiviral particles were injected into one hemisphere or the striatum, while the NCre-IntN-carrying lentiviral particles were injected into the other hemisphere. **b** In the hemisphere injected with the mTagBFP2-only lentiviral particles, no TdTomato signal was observed. However, in the hemisphere injected with NCre-IntN-carrying lentiviral particles, a TdTomato signal was observed, indicating the presence of functional Cre recombinase. Scale bar, 25 μm. **c** Venn diagram showing the percentage of cells expressing TdTomato. **d** Microscopic image showing the injection site of CTb-555 in the DS of wildtype mice. Scale bar, 200 μm. **e** CTb-555-labeled cells were observed in both the CeA and BLA. However, immunohistochemical analysis illustrated that only labeled cells in the CeA were GABAergic, while those in the BLA were not. Scale bars, 200 μm (10x) and 20 μm (40x). **f** Schematic diagram showing projection from CeA to DS. The FuG-E-NCre virus was injected into the DS of *Dlx6*-CCre-IRES-eGFP::LoxP-stop-LoxP-TdTomato mice, which was retrogradely transferred to the CeA. Cre recombination is expected to occur in GABAergic neurons in the CeA that project to the DS. **g** When FuG-E-NCre pseudotyped lentiviral particles were injected into the *Dlx6*-CCre-IRES-eGFP::LoxP-stop-LoxP-TdTomato mice, TdTomato cells were observed in the CeA. Scale bars, 200 μm (10x) and 20 μm (40x). **h** Venn diagram showing the percentage of cells expressing TdTomato in the CeA

First, we validated the reconstitution of split-Cre in our *Dlx6*-CCre-IRES-eGFP mice. We generated lentiviral vectors encoding either mTagBFP2 or NCre-IntN-mTagBFP2. We injected mTagBFP2 lentiviral particles as a control into one hemisphere of the striatum and injected NCre-IntN-mTagBFP2 particles into the other (Fig. 4a). While we did not observe any TdTomato expression in the hemisphere injected with mTagBFP2, robust TdTomato expression was evident in the hemisphere injected with NCre-IntN-mTagBFP2 (Fig. 4b). These results indicated that selective reconstitution of recombinatory Cre function occurred only when both NCre-IntN and IntC-CCre were expressed. Of the 37 neurons expressing both NCre-IntN and IntC-CCre, 78.3% exhibited a strong TdTomato fluorescence signal (Fig. 4c).

To selectively target long-range GABAergic projection neurons, we first validated the presence of these types of neurons that were found to project from the central amygdala (CeA) to the dorsal striatum (DS) [16] by injecting cholera toxin subunit B fused to Alexa Fluor 555 (CTb-555) into the DS of wild-type mice (Fig. 4d) [32]. We observed CTb-555 labeling in both the CeA and basolateral amygdala (BLA) (Fig. 4e). Immunohistochemistry analyses showed that only CTb-labeled neurons in the CeA were GABA-positive, whereas the CTb-labeled neurons in the BLA were not (Fig. 4e). This confirmed that only the projections from the CeA to the DS were from GABAergic neurons, as reported by Lingawi and Balleine [16]. Next, we generated retrograde lentiviral particles carrying NCre-IntN by substituting the vesicular stomatitis virus glycoprotein (VSV-G) with FuG-E, a pseudotyped lentiviral envelope where its fusion glycoprotein comprises of VSV-G and the rabies virus glycoprotein (RV-G) [12]. This allows NCre-IntN to be retrogradely transferred from the axon terminating in the DS back to the cell body of neurons in the CeA (Fig. 4f). Injection of the FuG-E-NCre-IntN viral particles into the DS of the *Dlx6*-CCre-IRES-eGFP::LoxP-stop-LoxP-TdTomato mice resulted in TdTomato-expressing neurons within the CeA (89.9% NCre- and CCre-expressing neurons expressed TdTomato) (Fig. 4g and h). While we observed NCre-IntN expression in some BLA neurons as the BLA also projects to the DS, we did not observe a TdTomato signal, indicating that Cre reconstitution was highly specific (Fig. 4g).

## Discussion

Here, we developed a split-Cre system based on split intein trans-splicing mechanisms. By generating a transgenic mouse model that expresses IntC-CCre in forebrain GABAergic neurons and NCre-IntN expressing retrograde viral particles, we could selectively target long-range GABAergic neurons projecting from the CeA to the DS (Fig. 4g). In the *Dlx6*-CCre-IRES-eGFP mouse generated, we noted only 83.1% of the IntC-CCre neurons were GABA-positive. This discrepancy could have arisen from our conservative approach in quantifying GABA-positive signals (2 standard deviations away from background signal) and the relatively thick 40 μm tissue preparation that could lead to poor antibody penetration and underestimation of GABA-positive neurons. We are still puzzled by the slightly lower recombination efficiency of our split-Cre system upon viral delivery schemes in vivo (approximately 80%, compared to 99% upon transfection; Fig. 4c and h), which could simply be due to the low titer (3E8 IU/mL) of our lentiviral particles. However, this result also indicates the selectivity and stringency of our split-Cre recombinase, since germline recombination and transient unwanted expression of Cre recombinase have been reported in even well-established transgenic mouse lines [28]. Given that we can genetically target long-range GABAergic neurons with high specificity, we can further investigate the functions of long-range GABAergic projection neurons during active behavior using existing Cre-dependent opto- and chemogenetic tools. In addition, our approach can be used to understand whether the projection patterns of a neuron correlates with its gene expression profile using RNA sequencing. Our approach should be able overcome the limitation of having few virally packageable, tissue-specific promoters [23].

Taken together, our combinatory split intein-mediated split-Cre system allows us to limit Cre recombinase expression to specific subsets of neurons by means of its gene expression profile and anatomical connections, enabling the systematic study of specific types of neurons in the mammalian brain.

## Methods

### Computer simulation of split-intein split-Cre recombinase product

Model for split-intein mediated split-Cre recombinase-LoxP DNA complex was calculated using automated modelling mode in the SWISS model server (http://swissmodel.expasy.org/), based on the crystal structure of Cre-LoxP synaptic complex (1NZB) as a template [5]. Split inteins is expected to form stable β-turn structure without any substantial structural changes in the original Cre recombinase. Figures for the structure were prepared using PyMol software [3].

### Construction of NCre-IntN and IntC-CCre

NCre and CCre were generated from the pCAG-Cre:GFP, a gift from Connie Cepko (Addgene plasmid #13776; https://www.addgene.org/13776/; RRID:Addgene_13,776) using the KAPA HiFi PCR kit (Roche Sequencing). IntN and IntC were amplified from the Npu DnaE inteins with site directed mutagenesis according to Lockless and Muir [18]. All plasmids were subcloned into a pCDH expression vector (System Biosciences), assembled using Gibson Assembly (New England Biolabs) and verified by Sanger DNA sequencing.

### Western blot analyses

HEK293T cells in 12-well culture plates were transfected with 1μg of each construct and Lipofectamine 2000 (Thermo Fisher Scientific) for 18 h. Cells were supplied with fresh media after transfection and grown for another 2 days. Cells were mechanically lysed in RIPA buffer containing phosphatase and protease inhibitors. The proteins were separated by SDS-PAGE under reducing conditions and transferred to polyvinyl difluoride (PVDF) membranes (Millipore, Billerica, MA). Antibody incubations were performed in 3% BSA in TBS buffer using the following antibodies: anti-HA, 1:1000 (MilliporeSigma); anti-FLAG, 1:1000 (MilliporeSigma); anti-β-Tubulin, 1:1000 (Covance).

### HEK293T in vitro reporter assay

HEK293T cells were seeded into 12-well culture plates containing coverslips treated with poly-L-lysine and transfected the next day with 1 μg of each construct using Lipofectamine 2000 (ThermoFisher Scientific) for 18 h. The cells were supplied with fresh media after transfection and subsequently fixed with 4% (w/v) paraformaldehyde in PB 24 h later. The coverslips were mounted onto microscope slides with FluorSave mounting medium (Calbiochem) and imaged with the Zeiss LSM780 confocal microscope (Zeiss Microscopy, Munich, Germany) using the 40x oil objective lens.

### HEK293T luciferase assay

HEK293T cells were seeded into 96-well culture plates and transfected with 1 μg of each constructed the next day. 24 h post transfected, we washed the cells with PBS and treated them with lysis buffer (Pierce Firefly Luciferase Glow Assay Kit, Thermo Fisher Scientific) for 20 min. The cell lysate was then collected and spun down to remove cell debris. 15 μl of the cell lysate was transferred into a 96-well black polysterene plate (Corning Costar). 45 μl of the working buffer (D-luciferin and the Firefly Glow Assay buffer according to manufacturer’s instructions, Pierce Firefly Luciferase Glow Assay Kit, Thermo Fisher Scientific) was added to the cell lysate and we waited 10 min before acquiring signals using a microplate reader (Tecan, Männedorf, Switzerland).

### Animals

Animals were housed in a specific pathogen-free facility maintained below 22 °C and 55% humidity under a 12 h light-dark cycle (lights on at 0700 h) with ad libitum access to food and water. All experimental procedures were conducted in accordance with guidelines for the care and use of laboratory animals for scientific purposes with approved protocols from the Institution Animal Care and Use Committee of SingHealth Research (Protocol number: 2014/SHS/921).

### In utero electroporation

Floxed Tdtomato transgenic females were time mated. On embryonic day 16.5 (E16.5), ex vivo electroporation and organotypic brain slice culture was performed as described elsewhere [17]. A GFP expression plasmid was used as a negative control, and a native Cre recombinase plasmid was used as a positive control. For the experiments, embryos were electroporated either with CCre plasmid only or with both NCre and CCre plasmids. For image acquisition, on day 3 of culture, the organotypic slice inserts were transferred to a 50 mm petri dish containing culture medium and imaged using an inverted long distance fluorescent microscope.

### Generation of DLX6-IntC-CCre-eGFP mice

We used the bacterial artificial chromosome (BAC) transgenic approach to generate the animals that express IntC-CCre under the DLX6 promoter. In this experiment, we constructed a knock-in cassette containing a FLAG-tagged (at the N-terminal of) IntC-CCre coding sequence followed by an IRES-eGFP cassette. A SV40 polyadenylation signal sequence was placed at the 3′ end of the cassette to stabilize the transcript. A FRT flanked Kan/Neo selection cassette was attached to the 3′ end of the IntC-CCre cassette to facilitate screening of the recombinant clones. We used recombineering technology to integrate the IntC-CCre expression cassette into the BAC clones containing the Dlx gene locus. In the recombinant BAC clones, the translation start codon ATG of FLAG-IntC-CCre is fused to the ATG of the endogenous Dlx gene. After deletion of the selection cassette by expression of Flp, the resultant BAC clones containing the IntC-CCre cassette were used to create the transgenic mice by standard pronuclear injection.

### Virus preparation

HEK293T cells were cultured in T75 flasks and transfected with hSyn.NCre-IntN.T2A.mTagBFP2, envelope and VSV-G or FuG-E packaging plasmid. pCAGGS-FuG-E was a gift from Dr. Kazuto Kobayashi (Addgene plasmid #67509; http://n2t.net/addgene:67509; RRID:Addgene_67,509). 18 h after transfection, we supplied fresh medium and the cells were further incubated for 48 h. The medium was collected and filtered through a 0.45 μm Minisart syringe filter (Sartorius Stedim, Goettingen, Germany). Viral vector particles were pelleted via ultracentrifugation at 25,000 g for 2 h. Supernatant was subsequently removed and the pellet was resuspended in sterile PBS overnight. Virus titre was quantified using the qPCR Lentivirus Titration Kit according to manufacturer’s directions (Applied Biological Materials, British Columbia, Canada).

### Stereotaxic injection of virus and cholera toxin subunit B

Mice were anesthetized with 2% isoflurane in oxygen at a flow rate of 0.5 L/min. Mice were placed in a stereotaxic apparatus to receive bilateral infusions of the virus or 0.5% (w/v) cholera toxin subunit B (CTb) Alexa Fluor 555 Conjugate (Invitrogen) into the dorsal striatum (+ 0.1 mm antero-posterior, 2.0 mm mediolateral, 2.7 mm dorsoventral); coordinates relative to bregma (Paxinos and Watson, 2007). 1ul of virus or 0.1 μl of CTb-555 was infused per hemisphere with a 30-gauge needle (Hamilton, United States) over 5 min and 0.5 min respectively using a programmable infusion pump (Hamilton, United States). The needle was left in the place for 5 min to allow diffusion of the virus or CTb before removal at 0.1 mm/sec. Following surgery, mice received 5 mg/kg carprofen intraperitoneally. Mice were sacrificed 28 days or 7 days following virus or CTb injection respectively.

### Perfusion and immunohistochemical analyses

Mice were anesthetized with isoflurane and transcardially perfused with ice-cold PBS, followed by 4% (w/v) paraformaldehyde (PFA) in 0.1 M PB. Extracted brains were submerged in PFA for an hour before transferring to 30% (w/v) sucrose in 0.1 M PB for cryoprotection. After 48 h, brain were sectioned at 40 μm and stored in PBS-Azide. Sections were first permeabilized in 0.2% Triton-X100 in PBS (30 min, RT) and later incubated in (i) a blocking solution of in PBS containing 5% donkey serum, 3% bovine serum albumin and 0.2% Triton-X100 (1 h, RT); (ii) the same solution containing the primary antibody overnight at 4 °C (anti-tRFP, 1:1000, Evrogen; anti-GFP, 1:1000, Abcam; anti-GABA, 1:2000, MilliporeSigma; anti-parvalbumin, 1:2000, Abcam; anti-somatostatin, 1:1000, Peninsula Laboratories) (iii) the same solution containing a 1:500 dilution of secondary antibody (Alexa Fluor, ThermoFisher; 2 h, RT). All sections were then stained with DAPI (1:5000 diluted in 0.2% Triton X-100 in PBS, Sigma-Aldrich), washed in 0.2% Trixton-X100 in PBS and mounted with FluorSave (Merck Millipore). Images of the immunostained sections were acquired using the widefield (Nikon Instruments, Tokyo, Japan) or confocal microscope (Zeiss Microscopy, Munich, Germany).

### Statistical analyses

Data were analysed and plotted using Estimation stats/plots [7]. *p* < 0.05 was considered statistically significant.

## Supplementary information

**Additional file 1: Figure S1.** Immunohistochemistry performed in 6 month old *Dlx6*-CCre-IRES-eGFP mice. S1(A-C) Microscope images of immunohistochemistry performed in 40 μm thick coronal sections using antibodies against Calcium/calmodulin-dependent protein kinase II (CaMKII), excitatory neuron marker (A), choline acetyltransferase (ChAT), cholinergic neuron marker (B) and glial fibrillary acidic protein (GFAP), astrocyte marker (C). Arrows indicate Dlx6-CCre-positive neurons based on GFP fluorescence do not overlap with either CaMKII, ChAT or GFAP signals. Scale bars: 25 μm in panel A, 50 μm in panels B and C.

**Additional file 2: Figure S2.** Brain sections of 6 month old *Dlx6*-CCre-IRES-eGFP mice showing normal gross brain structure. S2(A) A sagittal brain section of *Dlx6*-CCre-IRES-eGFP mice stained with DAPI. Scale bar: 1 mm. S2(B) Coronal forebrain sections of *Dlx6*-CCre-IRES-eGFP mice taken approximately 1 mm apart, stained with DAPI. Scale bar: 1 mm.

**Additional file 3: Figure S3.** Quantitative analyses of a 10 min open field test performed using 1 year old *Dlx6*-negative::Rosa26-LoxP-Stop-LoxP-TdTomato (littermate control [Dlx6- RTM]), and *Dlx6*-CCre-IRES-eGFP::Rosa26-LoxP-Stop-LoxP-TdTomato (Dlx6+ RTM). S3(A) Total distance travelled in open field chamber (cm), (B) Number of movements, (C) Number of stereotypy, which is the amount of repetitive movements, includeing grooming behaviour, (D) Time spent in center (s). We noted statistically non-significant differences (Mann-Whitney test) between Dlx6- RTM and Dlx6+ RTM in locomotor activity (*p* > 0.9999), movements (*p* = 0.9143), stereotypy (*p* = 0.2571) and time in center (*p* = 0.2571).

## Data Availability

Please contact author for data requests.

## Abbreviations

BAC: Bacterial artificial chromosomes
BLA: Basolateral amygdala
CCre: C-terminal Cre fragment
CeA: Central amygdala
CTb: Cholera toxin subunit B
DNA: Deoxyribonucleic acid
DS: Dorsal striatum
FuG-E: Fusion glycoprotein type E
GABA: Gamma aminobutyric acid
HEK293T: Human embryonic kidney 293 cells expressing mutant version of SV40 large T antigen
IntC: C-terminal intein
IntN: N-terminal intein
NCre: N-terminal Cre fragment
PV: Parvalbumin
RNA: Ribonucleic acid
RV-G: Rabies virus glycoprotein
SST: Somatostatin
VSV-G: Vesicular stomatitis virus glycoprotein
