# Supplementary material for: Neural circuit analysis using a novel intersectional split intein-mediated split-Cre recombinase system
Source: Mol Brain. 2020 Jul 2;13:101. doi: 10.1186/s13041-020-00640-2 (PMC7331137; doi:10.1186/s13041-020-00640-2)
Supplement: Supplementary file 1 [file 13041_2020_640_MOESM1_ESM.docx]

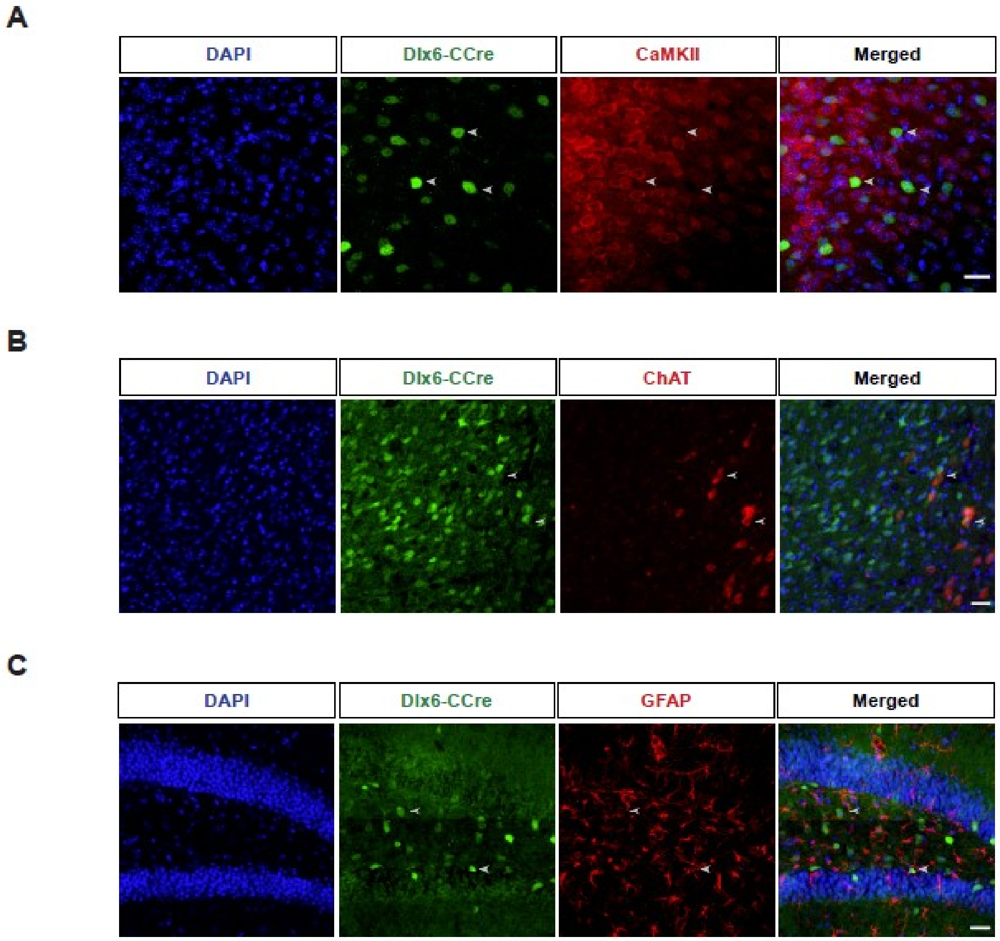
A
DAPI
Dlx6-CCre
CaMKII
Merged
B
DAPI
Dlx6-CCre
ChAT
Merged
C
DAPI
Dlx6-CCre
GFAP
Merged
