# Supplementary material for: Neural circuit analysis using a novel intersectional split intein-mediated split-Cre recombinase system
Source: Mol Brain. 2020 Jul 2;13:101. doi: 10.1186/s13041-020-00640-2 (PMC7331137; doi:10.1186/s13041-020-00640-2)
Supplement: Supplementary file 2 [file 13041_2020_640_MOESM2_ESM.docx]

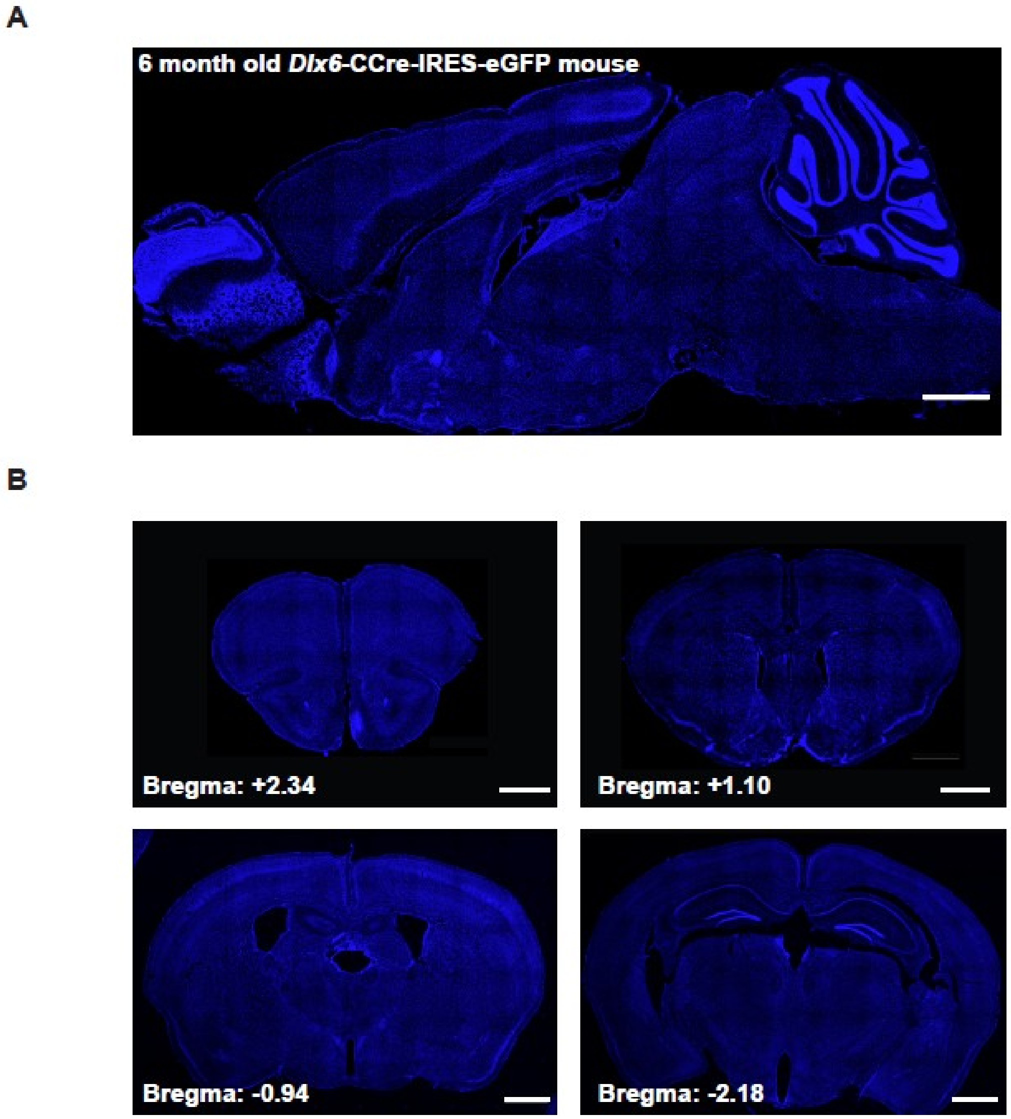
A
6 month old *Dlx6*-CCre-IRES-eGFP mouse
B
Bregma: +2.34
Bregma: +1.10
Bregma: -0.94
Bregma: -2.18
