# Supplementary material for: Neural circuit analysis using a novel intersectional split intein-mediated split-Cre recombinase system
Source: Mol Brain. 2020 Jul 2;13:101. doi: 10.1186/s13041-020-00640-2 (PMC7331137; doi:10.1186/s13041-020-00640-2)
Supplement: Supplementary file 3 [file 13041_2020_640_MOESM3_ESM.docx]

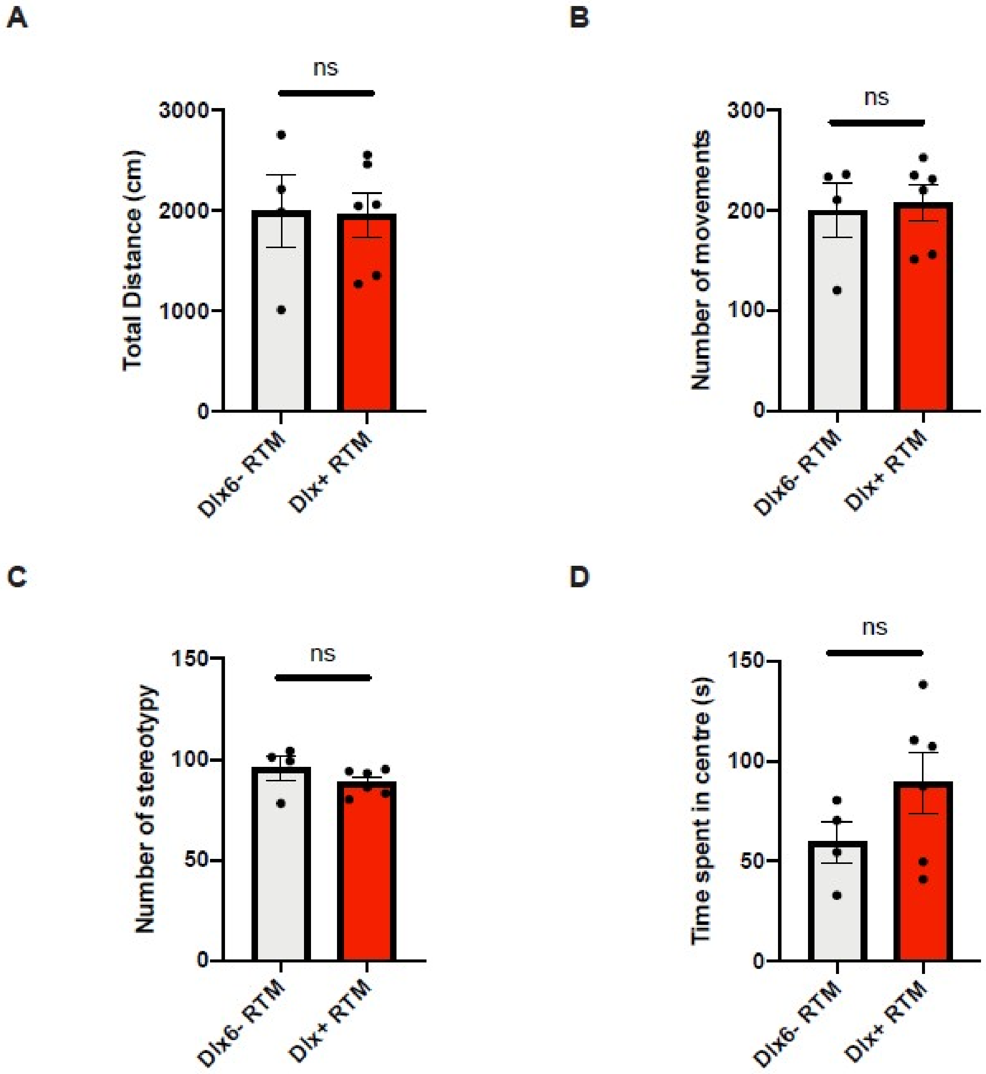
A
ns
Total Distance (cm)
3000
2000
1000
0
Dlx6- RTM
Dlx+ RTM
B
ns
Number of movements
300
200
100
0
Dlx6- RTM
Dlx+ RTM
C
ns
Number of stereotypy
150
100
50
0
Dlx6- RTM
Dlx+ RTM
D
ns
Time spent in centre (s)
150
100
50
0
Dlx6- RTM
Dlx+ RTM
